# Cardiac disease in pregnancy and the first year postpartum: a story of mental health, identity and connection

**DOI:** 10.1186/s12884-022-04614-1

**Published:** 2022-05-02

**Authors:** Jane Hutchens, Jane Frawley, Elizabeth A. Sullivan

**Affiliations:** 1 School of Public Health, Faculty of Health, University of Technology Sydney, Ultimo, NSW Australia; 2 Faculty of Health and Medicine, University of Newcastle, University Dr, Callaghan, NSW 2308 Australia

**Keywords:** Pregnancy, Postpartum, Mental health, Isolation, Connection, Self-identity, Cardiac, Qualitative

## Abstract

**Background:**

Women with cardiac disease in pregnancy and the first year postpartum often face uncertainty about their condition and the trajectory of their recovery. Cardiac disease is a leading cause of serious maternal morbidity and mortality, and the prevalence is increasing. Affected women are at risk of worsening cardiac disease, chronic illness, mental illness and trauma. This compounded risk may lead to significant and long-term negative outcomes. The aim of this study is to correct the lack of visibility and information on the experiences of women with cardiac disease in pregnancy and the first year postpartum.

**Methods:**

A qualitative study using in-depth semi-structured interviews with twenty-five women who had acquired, congenital or genetic cardiac disease during pregnancy or the first year postpartum. Data were analysed and interpreted using a thematic analysis framework.

**Results:**

Analysis of the interviews produced three major themes: 1) Ground zero: index events and their emotional and psychological impact, 2) Self-perception, identity and worthiness, and 3) On the road alone; isolation and connection. There was a narrative consistency across the interviews despite the women being diverse in age, cardiac diagnosis and cardiac health status, parity and timing of diagnosis. The thread prevailing over the temporal and clinical differences was one of distress, biographical disruption, identity, isolation, a necessitated re-imagining of their lives, and the process of multi-layered healing.

**Conclusion:**

Acknowledging and understanding the breadth, complexity and depth of women’s experiences is fundamental to improving outcomes. Our findings provide unique insights into women’s experiences and challenges across a spectrum of diseases. Most women did not report an isolated trauma or distressing event, rather there was a layering and persistence of psychological distress necessitating enhanced assessment, management and continuity of care beyond the routine 6-week postpartum check. Further research is required to understand long-term outcomes and to refine the findings for specific disease cohorts to be able to respond effectively.

## Background

Cardiac disease is a leading cause of maternal morbidity and mortality [1, 2]. The term ‘cardiac disease in pregnancy and postpartum’ (CDPP) describes a heterogeneous group of acquired, congenital and genetic conditions, including structural heart and aortic disease, cardiomyopathies, rhythm disorders, ischaemic heart disease, and arterial dissections. These conditions are pre-existing, or diagnosed in pregnancy or the postpartum period.

Individual cardiac diseases in pregnancy may be rare; however, international prevalence estimates for combined CDPP are 1% to 4% [1] with evidence of increasing prevalence [1, 3]. In Australia, the overall maternal mortality rate is low and stable; for 2015–2017 cardiovascular disease was the second most common cause of death, accounting for 13.6% of deaths [4]. CDPP is associated with serious maternal morbidity across physical, psychosocial and functional domains. An estimated one in four women with cardiac disease during pregnancy requires hospitalisation [2] and for each maternal cardiac death nearly eight women have severe morbidity [5].

Current estimates of prevalance and mortality, and the scale and nature of morbidity, are likely to under-ascertain the disease burden. Morbidity monitoring is affected by inconsistent definitions and criteria, language and monitoring practices [6, 7]. Newly defined conditions may not be captured in earlier research, and diagnosis may require a level of imaging equipment and expertise not available at all sites. Additionally, both mortality and morbidity studies inconsistently include women diagnosed in the late and very late postpartum periods [8], women managed outside the hospital system, and women with milder symptoms who go undiagnosed. This limits our understanding of the breadth of disease characteristics.

Based on a prevalence of 1% to 4%, there are 1.3 to 5.2 million women affected annually by CDPP globally, of which 3,150 to 12,600 are Australian. The lack of research exploring the experiences of this considerable cohort of women, is in and of itself, a reason for further inquiry.

There is a lack of comprehensive data on the experiences of women with CDPP. In addition, only a limited number of studies have reviewed the impact of CDPP on activities of daily life, mental health, quality of life, relationships, loss of income, and the effects of the persistent risk of worsening cardiac health and premature death. A recent meta-synthesis of 11 studies that examined the experiences of women with existing or acquired CDPP who were or had been pregnant, or who had contemplated pregnancy confirmed the paucity of research about women’s experiences and highlighted the need for women to share their stories [9]. These stories could inform the development of models of care that are responsive to women’s needs, values, knowledge and desired outcomes.

Women with CDPP are situated at the intersection of several and varied medical and psychological conditions, each potentially bringing significant morbidity. They are at risk of worsening cardiac disease, chronic illness, mental illness, trauma and death. Their pregnancy, labour, birth and postpartum recovery may be complicated. This compounded risk exposure may lead to significant and long-term negative outcomes. It is critical to understand women’s experiences to develop and provide evidence-based guidelines that meet their needs in the acute hospital setting and after discharge to the community.

The aim of the study is to explore in-depth the lived experiences of Australian women with cardiac disease in pregnancy and the first year postpartum.

## Methods

### Study design

A qualitative study design using in-depth semi-structured interviews was designed to achieve the aim of understanding women’s experiences of cardiac disease in pregnancy and the first year postpartum. Qualitative health research provides an insight into patient experiences that not only validates their stories but also raises awareness, and makes meaningful change possible [10]. The concept of the study was discussed with clinical and community groups from the NSW Heart Foundation.

## Participants

Women with CDPP who gave birth in Australia to at least one live born baby of 20 weeks’ gestation or 400gm birthweight were invited to participate. CDPP is defined as pre-existing or newly diagnosed cardiac disease in pregnancy or in the first 12 months postpartum. Women required adequate English fluency to participate in the interview and women whose primary diagnosis was hypertension, pre/eclampsia or thromboembolic disorders were excluded from the study.

Recruitment was via advertisements posted on the social media of selected consenting pregnancy and parenting groups and cardiac support groups, and via direct and indirect invitations distributed by cardiac support groups to members’ emails and or group newsletters. Participants were purposively recruited because they are, or have been, mothers with a diagnosis of cardiac disease who were willing to participate in an in-depth interview. Recruitment continued until we had adequate depth and breadth of data to sufficiently describe and analyse the participants’ experiences and answer our research question [11]. Recruitment was from December 2018 to April 2020.

## Data collection

We used semi-structured, in-depth interviews which is a recognised qualitative approach for investigating topics about which little is known and it privileges the issues that are meaningful for the participant and can accommodate diverse perceptions [12]. Individual interviews conducted by phone and were conducted by a single interviewer (JH). Interviews began with confirmation of informed consent and the collection of basic demographic data. Participants were asked to choose a pseudonym to protect their identity. The interviews took between 24 and 90 min and with the women’s permission interviews were audio-recorded or hand-transcribed verbatim, including notable non-verbal responses such as crying or laughing.

An interview guide was developed with the lead question “Can you tell me about your experience?” Additional open-ended questions were formulated during the interviews in response to the participant’s story and where appropriate, prompts were used to clarify and expand on the account provided. This approach enabled participants to share any information they wanted to, including feelings, attitudes and reflections in their own words, and to provide a space that encouraged them to direct the narrative of their story.

### Data management and analysis

Interpretive inductive thematic analysis was used as it is flexible and responsive when unexplored phenomena are described; facilitated the coding and organisation of a large and complex data set and nuanced theme generation; and was suitable for rich narrative description [13, 14]. Informed by the six stages of analysis outlined by Braun and Clarke [14, 15] (familiarisation, code generation, theme development, reviewing and refining themes, defining themes and report writing) data coding and preliminary theme generation occurred concurrently with the interview fieldwork and was iterative and responsive to new data and developing patterns.

All study team members listened to the interviews and read the transcripts. JH led the analysis by immersing herself in the data and developing and refining codes and themes and selecting illustrative quotations. The approach to analysis was broadly informed by critical theory and social constructionism with latent themes (reflecting the underlying assumptions and meanings of the stories and semantic meanings) [15, 16].

### Ethical considerations

Written or recorded verbal informed consent was obtained from all participants. Ethics approval was granted by the University of Technology Sydney’s Human Research Ethics Committee (ETH19-3372).

## Results

We interviewed twenty five women of different ages who had a variety of diagnoses and timing of diagnosis, but who all had a rare, potentially life-threatening cardiac condition whilst pregnant or postpartum. The majority lived in metropolitan areas, and of the four who lived in regional or rural areas, two transferred to metropolitan hospitals for care during their pregnancy or postpartum event. More than half had tertiary level education (60%), seven (28%) had trade level and three (12%) had high school education. Their median age at interview was 39 years (range: 28–59) and age at time of diagnosis ranged from 2 days to 46 years old. Their cardiac diagnoses were categorised as congenital (*n* = 5), genetic (*n* = 9) and acquired (*n* = 12) conditions, with one woman having both genetic and congenital diagnoses. Timing of diagnosis was prior to pregnancy (*n* = 9; diagnosed from 2 days old to 26 years old), antepartum (*n* = 6) and postpartum (*n* = 10; diagnosed from 2 weeks to 11 months postpartum). Most were first-time mothers (*n* = 15), five had their cardiac diagnosis associated with the 2^nd^ birth, four with their 3rd birth and one with her fourth birth. Six women had subsequent pregnancies. There were no stillbirths or neonatal deaths for the pregnancies discussed. Participant diagnoses are outlined in Table 1.

**Table 1:** Participant diagnoses

**Congenital heart disease**
Bicuspid Aortic Valve (BAV)
Left Ventricular Non-Compaction Syndrome (LVNCS)
Mitral Valve Prolapse (MVP)
Patent Ductus Arteriosus (PDA)
Patent Foramen Ovale (PFO)
Tetralogy of Fallot (TOF)
**Genetic**
Arrhythmogenic Right Ventricular Dysplasia (ARVD)
Hypertrophic Cardiomyopathy (HCM)
Long QT Syndrome (LQTS)
**Acquired**
Idiopathic Cardiomyopathy (ICM)
Peripartum Cardiomyopathy (PPCM)
Pregnancy Associated Spontaneous Coronary Artery Dissection (PSCAD)

The interviews provided complex and varied data. Analysis produced three major themes: 1) Ground zero; index events and their emotional and psychological impact; 2) Self-perception, identity and worthiness; 3) On the road alone; isolation and connection. The themes and sub-themes are described as discrete entities, however they overlapped, and interacted with each other. Movement between themes and stages was fluid and iterative, and not a linear trajectory from illness to health.

## Theme 1: Ground zero: index events and their emotional and psychological impact

Women across the spectrum of diagnoses described traumatising events, feeling disempowered, experiencing psychological distress and struggling to recover emotionally.

## Shock and anger

Women were shocked and distressed by their clinical status and by the way it was communicated to them. One woman attended a routine cardiac review feeling well and unchanged in her health and was unexpectedly told:

“Your heart is so bad you’ll need a transplant at some point” … oh, and then he said, “Hmmm, that’s if you get a heart.” (Woman 20)(W).

Some women felt angry that they had a serious cardiac condition that imposed significant restrictions on them, especially given they had healthy lifestyles and no traditional cardiac risk factors: “It just felt really unfair and I just felt really sad”.(W9). The frustration of not being able to do what they once did merged the anger and the anxiety, “I was so angry… and I’ve still got the demons (anxiety).”(W7). Women were also angry and “pissed off” (W25) that their condition or deterioration was not diagnosed earlier despite multiple presentations to doctors and emergency departments, where they were repeatedly dismissed.

## Anxiety and post-traumatic stress disorder

Anxiety was common and for some women symptoms of anxiety were pervasive and unrelenting. Triggers were varied, including concerns regarding access to care, “…anxiety-wise … it’s always in the back of your mind that if something does happen, then we are kind of screwed” (W1), and a lack of trust in the health professionals; “I was also fairly panicked thinking, ‘I don’t feel confident you know what’s going on’”. (W23).

## Fear and anxiety were also in direct response to cardiac events or deterioration

I just fel﻿t everything fading away. And I was really, really scared. I got again that impending sense of doom, I just, I thought, I’m dying, I’m dying. (W25).

Women were alarmed that they nearly died; that they unknowingly had a condition that “could have killed [them] at any point” (W12) and that when they ‘Googled’ their condition the main information accessed exacerbated their fears, “Oh God, you just think you’re going to die”. (W25).

Women with persistent cardiac symptoms, especially if they were the same as for their major cardiac event, had a seemingly unremitting trigger for anxiety.

*[Pain]… several times a day feeling like, “Oh, is this it?” [worrying they’ll] drop dead every five, ten times we have the pain a day. (W16).*

They described feeling “stunted by fear and anxiety” (W12), and not attending rehabilitation or exercising due to fear of triggering further cardiac damage.

I was scared out of my wits, and I got to the stage where I couldn’t push the pram for fear that if I had [another] heart attack, the pram would be pushed under traffic or something. I wasn’t only fearful for myself, it was more for the baby. (W16).

Women with genetic conditions had the additional worry about passing on their conditions, “the anxiety that I felt around thinking that my son had it was so overwhelming.” (W12). Several women had persistent intrusive thoughts from their “very traumatic experience”. (W23).

You’ve just got these constant daily reminders … I feel like that’s all I’m ever thinking about. So unless I’m distracted I find it quite hard to be positive now. And yeah, it’s definitely taken a toll. (W9).

Trauma was not necessarily an isolated incident, instead “…there’s sustained trauma in it. There’s trauma from the start, and then there’s just trauma”, (W12) and a number of the women required treatment for post-traumatic stress disorder (PTSD). Typically, women experienced prolonged or repeated distress and felt unsupported, which compounded prior experiences, creating multiple points of harm. For example, one woman who felt physically weak and dizzy, was traumatised and distressed by her birth experience, and whose baby was in the neonatal intensive care unit (NICU) asked for help to be taken to the NICU after they called for her and was told:

“Oh, no, no. Just go down there and if you feel like you’re going to pass out, sit down on the floor and just yell out and someone will hear you.” (W14).

The fear, trauma and “horrid postnatal depression” she experienced made her determined to not return to the hospital where it occurred however she was transferred there when ill a number of years later.

“I’m not going back to that major hospital because I’m absolutely traumatised. I never want to go there ever again for any reason, ever,” and I have been there since, and I had an absolute panic attack … They opened the door of the lift … right next to NICU, and I lost my mind. I absolutely lost it, I was crying and shaking. It was … a trauma response. (W14).

## Multifaceted, compounded trauma, grief and depression

Women ‘lost months’ where they were on emotional and cognitive auto-pilot. It took time to recognise the level of anxiety, terror and depression that they had been living with. For some women it wasn’t until weeks, months or years later that they experienced generalised or postnatal depression or when “…it all sort of came unravelling”. (W21) For others and their families, the emotional toll of their experiences did not come into focus until their physical health stabilised.

…as I started to get a little bit physically better was probably when …mentally and emotionally things started to fall apart for me. (W9).

The negative impact on their mental health was long-term for some women and for their families.

I think it is only years later that the toll has become very evident, for the whole family, in terms of mental health. I think the whole family has struggled with it … That’s the fallout really. (W20).

The experiences of trauma, grief, anxiety and depression were layered and the impact of the complex array of losses was at times exacerbated by the communication style of health professionals. Most women were advised to avoid future pregnancies; the grief and distress felt in response to this advice was compounded by their loss of autonomy in decision-making about such a fundamental issue in their lives.

[I see the Dr] …two minutes each day doing rounds, and he goes to me “Oh, that means no more babies too now”. It was just such a blasé comment, and then he left. That was sort of a bit of a devastating blow at the time too because I would have liked to have made the decision … I took that really hard. (W10).

They grieved previous pregnancy loss and loss of time with their newborn and older children as they struggled in the acute aftermath of their experiences and because they were restricted in what they were able to do in 28.

I could not believe that it wasn’t enough that I’d lost a child, that I had to go through [this] shit on top of it. (W7).

## Recognition, support and healing

Following their cardiac event or birthing experience women felt alone, isolated, and that their mental health needs were almost exclusively not recognised.

You know, year after year, and not getting any help. No-one saying, “Well, maybe you should talk to your doctor about depression.” (W16).

All but one woman who had counselling, sought it out themselves. Psychological recovery has been of an uneven pace and trajectory and was informed by their individual histories, situations and support. Ongoing symptoms and undesirable cardiac outcomes complicate efforts to heal emotionally and for some women “the hardest part of recovery is the emotional recovery” (W7) which was invisible and more difficult and protracted than physical healing.

I have really struggled with it – it’s been a year and I’m still kind of, you know, grappling with all that sort of stuff. (W9).

… my heart’s healed; I still have the psychological healing to do. (W15).

Recovery from the psychological impact of their experiences required that they were able to carve out some space and time to care for themselves, to centre their mental health. Women consulted with psychologists and counsellors, incorporated meditation practice, and connected with other women with similar experiences.

Stigma surrounding mental health, a self-perception of not needing help, and a tendency to put other people’s needs first influenced women’ reluctance to seek help. Several participants recommended that mental healthcare be incorporated into routine post-cardiac care and clinical guidelines to overcome barriers to access.

You put yourself last. If you have something that’s told you’re supposed to go do [de-briefing, counselling], then you might make the time for it. (W15).

The women identified that they required specialised psychological care and that “medical professionals who deal with physical health … just don't seem to be very aware of the psychological aspect.” (W13).

So, physical care is one thing, but I think anything around mental health in it is just critically important. Because that’s the thing that’s either going to get you through the rest of your life or not, or hold you in a space that you just won’t be able to move from …this experience for me wasn’t very long ago, and I don’t feel I was offered nearly enough assistance around that. (W12).

Connecting with peers through support groups or a dedicated peer support program was also identified as being valuable.

Talking to someone who’s the same age who’s done the same kind of things, or is at the same life level with the same conditions, is really helpful. (W1).

## Theme 2: Self-perception, identity and worthiness

Becoming a mother and having a major cardiac event brought changes to women’s self-perception, identity and understanding of what their lives will be.

### Was it me? This is not me.

In the immediate period after a shock it is common to question if you were responsible or somehow contributed, as some women in this study did, “all of a sudden I’m thinking, God, I ate too many hot chips, you know, I didn’t exercise enough” (W15). However, this extended to self-deprecation, “I’m just so tired; it’s just ‘cause I’m old and fat” (W2) and blaming themselves for failing to detect changes in their condition that their specialists hadn’t, including pre-eclampsia “…it’s sort of a bit of ignorance on my behalf too…it’s my baby. (W21).

Women with pre-existing disease were well before their pregnancies and women with new diagnoses were well before their cardiac event, which made it hard to identify with having an acute event or new chronic health issue.

It’s when you’re very healthy and then you’re told that you’re not healthy … I didn’t feel sick, I didn’t look sick, I didn’t act sick, so it was really challenging for me to comprehend that. (W22).

Not identifying with a condition, especially once they felt well again, contributed to some women not wanting to join a support group that “… makes it more defining than what I want it to be”. (W17).

Some women identified as being “pretty strong and resilient” (W5) and of having a “level of emotional intelligence [that] is quite high” (W2); this was both their nature plus they had developed skills and resilience through previous experiences.

### Who am I now?

Women described the uninvited dismantling of existing self-identity as a consequence of their experiences and the need to re-shape their idea of who they were as they moved ahead.

“I was very very active, I was the manager of several [sites]. I’ve been a very keen athletic, sporty kind of person, so I was very, very healthy before. So it was a real lesson to have to change my whole being, change my whole lifestyle.” (W16).

When you’re diagnosed with something like this that you’ve always had, you’re sort of left feeling a bit empty and weird. You have to just keep going now, but you’re a different person … everything’s different. (W12).

### (Un)Deserving and downplaying

Women delayed seeking care or calling an ambulance, including mid-heart attack, because they thought someone else may be more deserving of that care or service. As they found their voices in recovery, several wanted to stress that women are worthy of healthcare and that they need to call the ambulance, to seek help.

Particularly as mums, we always put ourselves last and we think we’re wasting other people’s time. That’s why I refused the ambulance because I thought … someone could die because they’re with me, and you know, I’m not important and I’ll – I’ll be fine, I’m a fighter, I’m resilient, you know, I deal with things. And I think a lot of women are like that. We always put ourselves last, and it’s just so important not to do that. (W5).

In addition to the idea of being worthy of emergency medical help, women’s sense of worthiness was also demonstrated in comments about the impact on their families and not wanting to be a burden on them, “I feel like everyone’s already helping us out so much.” (W23).

Other times the question of worthiness was external, for example when the focus was on their partners and not them, making them feel disregarded and invisible.

“Oh, my God, your husband’s so amazing. He’s doing these great things with the kids.” I’m like, “Well, yes, I get it, but I nearly died here and I’ve got no choice and what about me hey?” (W7).

The women interviewed downplayed their medical condition or concerns and this was associated with fear and anxiety, guilt, regret, not wanting to upset or be a burden on their families, reluctance to take up space, the sense that they had somehow caused the condition and that they have given it to their children.

## Theme 3: On the road alone; isolation and connection

### Isolation and alienation

Women felt alone and isolated both within the healthcare system and socially. For women with very rare conditions “it is isolating and I've struggled quite a lot psychologically with it …I've never met anyone who has my condition or spoken to them.” (W13). When they were referred to general cardiovascular disease resources “it just felt alienating because it didn’t relate to me at all.” (W15). Many women longed for connection.

I felt very alone in those early years, and my God it would have helped me just knowing that there were other people. My cardiologist had told me at the time, about a week after my episode he had someone that had the same… I remember thinking, God it would be good to talk to her. (W6).

Women with pre-existing disease or who were diagnosed during pregnancy could not attend antenatal or parenting classes as they were categorised as being high-risk.

I’d love to have sat in on the parenting class … and I didn’t get to have a baby shower ‘cause I was in hospital. So, these little things … you don’t realise the social impact. (W2).

Women from across all diagnoses either could not attend mothers’ groups, or if they did the experience was uncomfortable, limiting the establishment of valuable peer mother networks.

They were all really freaked out … it was a very weird, isolating thing to go through. (W12).

## Making new connections

Support groups were only available for some of the cardiac conditions women were living with; for some conditions, there were no face-to-face or online support groups, for others there were groups for the general condition but not as it relates to younger people or women (e.g. cardiomyopathy). There were only international online groups for some of the cardiac conditions and some of the participants joined groups related to implantable defibrillators rather than the diagnosis that required the defibrillator. There were no support groups for women who had CDPP.

The women who read social media pages from the sideline may not have had the same level of engagement or benefited from the same level of support as those who did engage, instead having a vicarious support experience which they nonetheless described as helpful. Supports groups offered a space to connect, receive support and learn more about their diagnosis, including the psychological aspects of their condition and the day-to-day reality of living with a chronic illness. One woman had not met or communicated with anyone with the same diagnosis until she read a story about SCAD in a magazine and subsequently joined the online support group; this was 8 years after her PSCAD.

Women learnt more about their condition and management options in the groups, and so were better able to discuss their concerns and ask questions of their cardiologists. One woman found “connecting with people who have been in [her] situation” prior to having cardiac surgery was “incredibly valuable in putting [her] mind at ease.” (W3).

Online groups enabled varying degrees of engagement and meant women could connect at any time, without having to arrange childcare or transport; this was particularly helpful for women experiencing anxiety in the middle of the night.

I think without those support groups I really would have worried about my mental health. They have absolutely saved me. To be able to … write to people in the middle of the night and say “I’m feeling really scared. I can’t sleep. I’m gasping for air” – obviously I was having anxiety episodes, and people were saying “It’s okay. You’ll be okay. This is the same experience I had. This is what it’s all about.” (W12).

Some women found accessing the groups early to be helpful, whereas others said that would have been too distressing with a risk of vicarious trauma.

… the first year is really hard, and you might be frightened to death that every night you go to sleep you’re going to die, but we’re all here and we didn’t and it’ll get better. So it gives some hope. (W15).

I am kind of glad I didn’t find them early on because you do see some horror stories, and … that there is someone ten years on still experiencing heart attack pain. You don’t want to know that when you’re just out of hospital for the first time. (W16).

A downside of support groups is comparing yourself to others in an unhelpful way, “You risk comparing your treatments and that might not be appropriate because we’re all different” (W20) and this might “add a bit of fuel to the fire.” (W19). This was particularly relevant for international groups where women were receiving different advice and treatment, including being supported to have further pregnancies when Australian women were not, and this led to some women leaving those groups as it was too upsetting. Reading about other women’s experiences gave the women strength and was “quite humbling”. (W1). It was also “so depressing…everybody had such a hard time it was hard to digest” (W15).

While the groups provided a community and a space to share experiences, a weight of responsibility fell on the volunteer moderators, including logging on late in the evening to make sure women were not left feeling distressed and abandoned.

I have thought about the fact that someone might be sitting in bed in a hospital [in the middle of the night], feeling very isolated, and waiting for me to answer and join them to the page, so they could sit there and read. (W15).

Not all women joined groups because they were not on social media, don’t like group formats, because they did identify with the others in the group or their circumstances, or because they felt it was more helpful for them to find support elsewhere. For some, the issue was about not over-identifying or not wanting “… to get caught up in that space…it's already in my head enough, I don't need it in my head any more than that” (W17) or because “…not focusing on it 24 h a day is functional. It helps you get through sometimes.” (W2).

## Discussion

This study is an in-depth exploration of the lived experiences of Australian women with CDPP. Drawing directly on women’s stories, the analysis demonstrates that women with CDPP often have complex and distressing experiences that affect their sense of identity and mental health beyond their pregnancy or first year postpartum. Many of the experiences described by the participants are similar to those described in the literature on the lived experiences, mental health, self-identity and recovery of other populations such as cardiac, maternal, rare disease, chronic illness, trauma, birth trauma, severe maternal mortality and women with high-risk pregnancies [12, 17–20]. The women in our study were part of most, or all, of those populations making them uniquely exposed to multiple and compounded risks.

There was a narrative consistency across the interviews despite the women being diverse in age, cardiac diagnosis and cardiac health status, parity and timing of diagnosis. The thread prevailing over the temporal and clinical differences was one of psychological distress, biographical disruption, identity, isolation, a necessitated re-imagining of their lives, and the process of multi-layered healing. The women were resilient in their recoveries; however, it is important to not romanticise their post-traumatic growth as something other than difficult and demanded by circumstance.

## Mental health

Our research is consistent with previous findings that women diagnosed with CDPP describe feeling terrified, devastated, having a sense of doom, and feeling a loss of trust in the health system after having symptoms dismissed or misinterpreted health care providers as psychiatric symptoms (including “new mum anxiety”) [12, 21]. Many of the women in our study had been acutely unwell, some with life-threatening episodes, they had intense and or prolonged pain and significant decreases in functional capacity yet most described the mental health effects as having the greatest impact on them. Further, the lack of mental health and social support was reported to be at least as pronounced and harmful as the sparse amount of cardio-obstetric clinical research informing evidence-based practice.

CDPP is associated with significant mental illness. A recent study found 26 of 40 participants had a major mental health disorder diagnosed after experiencing peripartum cardiomyopathy (PPCM) [22]. Compared to postpartum women without PPCM, the prevalence of major depressive disorders was 4-fold, for post‐traumatic stress disorder 14‐fold, and for panic disorder 6-fold. Women of reproductive age with heart disease report feeling stress, having challenges in taking control and making decisions, experiencing a lack of autonomy, and the importance of social support is identified [9]; these characteristics are also determinants of perinatal anxiety [23]. Women with pregnancy-related spontaneous coronary artery dissection (PSCAD) are reported to have almost twice as high scores for anxiety and depression than men and non-pregnant women who had SCAD [17]. Further, a recent systematic review estimated the overall prevalence of perinatal depression to be 11.9% [24]. Mental health conditions, heart disease, and pregnancy and postpartum are closely interrelated.

Our findings on mental health and cardiac disease are disappointingly predictable and are evidence of a persistent lack of integrated mental health and social support for women with CDPP despite evidence of its need [17, 24]. In particular, younger women appear to be more exposed to the risk of reduced mental health [17] and some cardiac conditions, including those experienced by this population, have higher rates of associated mental health issues [17, 25]. The bi-directionality of mental health and cardiac disease means anxiety, depression and PTSD, as well as other mental health conditions, are associated with lower attendance at medical follow-up visits, poorer cardiac outcomes, more recurrent cardiac events and higher mortality [26–28].

Cardiac disease and suicide are leading causes of maternal mortality in middle- and high-income countries [4, 29, 30] though figures are likely to underestimate prevalence due to reporting differences and some data only including up to 42 days postpartum [29, 30]. A 15-year population study in Canada [31] is consistent with an earlier Australian study [32] in identifying a peak in suicide occurring between 9 and 12 months postpartum. Critically, compared to matched living women, perinatal women who died by suicide had a similar pattern of use of non-mental health primary care and obstetric care to women who do not suicide before the index date, reflecting a missed opportunity to intervene [31].

Risks factors for maternal suicide include a history of mental illness, indigeneity, lack of recognition of mental health, medical illness, poor inter-disciplinary communication and lack of continuity of care [4, 33]. The prevalence of self-harm, suicidality and anxiety are increasing in young women [34] and when they seek support for self-harm and suicidal ideation young women report feeling patronised and dismissed, and that emergency department (ED) experiences increase their risk of future self-harm [35]. Thus, some of the risks for maternal suicide are increasing without a concurrent increase in effective intervention prior to pregnancy or once a woman is pregnant or postpartum. Further, the experiences of young women being dismissed, the lack of recognition and lack of continuity of care mirror the experiences of the women in our study when needing mental health, cardiac and obstetric care, indicating a repeated pattern and compounding and growing risk for poor mental health outcomes.

## Autonomy and loss

In addition to the risk of delayed cardiac and mental health diagnoses, being dismissed and not receiving information when it was available reduced women’s agency and amplified the sense of low internal locus of control. Our findings were comparable to earlier research which reported that when women with maternal morbidity felt dismissed and not listened to, or when they didn’t fully understand the situation, they felt disempowered and a lack of health locus of control that persisted months after their experience [36, 37]. Consistent with previous studies, for women in our study feeling confident in their sense of self was most challenged when they were in acute medical situations and when they experienced medical gas lighting; the dismissal and invalidation of their sense of self and lived experience [38, 39]. The clinical risks of not being believed were compounded by the emotional distress these experiences resulted in.

Women may experience difficulty in making decisions and in having others making decisions for them [21, 40, 41]. Most women in our study were advised to cease breastfeeding and or to have no further pregnancies. Consistent with earlier research, women in our study described having no bodily autonomy and that this advice was most commonly issued as a directive, and not introduced as a discussion with the opportunity to discuss their desires and values [12, 21].

This loss of autonomy as well as previous subfertility and pregnancy loss, and the abrupt change to expected family size is disempowering and complicates grief. Perinatal loss, infertility and medical reasons to not have children are ambiguous losses, which are characterised by the simultaneous physical absence of, but psychological presence of the foetus or infant [42–44]. Ambiguous loss, for the children hoped for but never conceived, is associated with feeling frozen in grief, lack of support, and lack of recognition and rituals of grief. In the context of surviving a “concurring crisis” [45] of a complicated pregnancy or postpartum cardiac event this ambiguous and invisible loss may not be recognised by others including healthcare professionals and women may feel unable to talk about it [46]. The mental health impact of ambiguous loss may thus be complicated by disenfranchised grief in which a person experiences a significant loss and the resultant grief “is not openly acknowledged, socially validated, or publicly mourned” ([47]; p224) which exacerbates their suffering.

Being physically unwell with cardiac disease affects women’s ability to care for their newborn or return to the normal activities of daily life and these limitations contribute additional distress [12, 21]. Further, traumatic birth (as experienced by some of our participants) impacts a woman’s experience of motherhood and her initial relationship with her baby, with women reporting lost bonding time, feelings of failure and the emotional impact of ‘relinquishing care of the infant’ [18, 21]. Women struggle to surrender care even when acutely unwell, and the importance of their role as mother is not always fully appreciated or facilitated by health professionals [48]. Hinton, Locock, and Knight [49] highlight the difficulties of being a new mother in critical care, including the anguish of women who were separated from their baby, how important breastfeeding was for them, as well as the need for more support.

## Identity

Physical and mental illness, loss, and embarking on motherhood, especially for the first-time, provoke a changing sense of self and of self-identity [50–53]. Motherhood is a fracturing of a woman’s identity to allow space for a new identity in her new life [54]. This rupturing and reformation is a multidimensional process comprising the stages of triggering event (becoming a mother), loss of self, and redefining the new self; the women in our study repeated this process with the trigger event of CDPP. The women’s descriptions of cardiac and mental illness, pregnancy, birth and motherhood, identity and recovery in our study were congruous with Wisdom’s analysis relating to mental illness [54]. In this, participants described a loss of identity/self, the duality of being ill and well (of having cardiac disease and being a mother), perceptions of normality (the ‘new norm’), and their specific concerns about the impact on parenting, and recovery and reconciliation.

Pregnancy, birth, the postpartum period, breastfeeding and illness are fundamentally embodied experiences. The body is our access to perceiving and interacting with the world and is integral to identity and sense of self [55]. Biographical disruption is a disruption or disturbance of one's embodied perception and experience of the world [56]. A body changed by pregnancy and motherhood, and which is also now an unreliable body due to cardiac disease, complicates and unsettles existing identity and sense of self, and changes the way one interacts with the world. Illness (cardiac, mental, maternal) disrupts that which was taken for granted such as being able to pick up your baby, work, or play in your team sport, and causes a “fundamental re-thinking of a person's biography and self-concept” ([57]; p169). Moreover, chronic illness involves a continued adaptation, an ongoing struggle to “maintain control over the defining images of self and over one’s life” ([58]; p5). As their cardiac status, mental health and experiences of mothering change, women with CDPP have an enduring experience of re-defining self and identity, managing their illnesses and grief, and accepting and making sense of their lived experiences.

Self-identity is formed through reflection of our peers, and verified or rejected through our interactions with others, including healthcare professionals, through whom we internalise a felt sense of what it means to be ourselves, incorporating values and judgments that have been perceived [51, 59]. Mothers build their identity in part through engaging with peers in antenatal and mother’s groups that many of our participants were unable to join. People with illness compare themselves to others they know with the condition, or the knowledge they already had of the condition. Women change their self-perception post cardiac event, both positively and in the sense of losing one’s prior self [19]. Most women in our study had no, or few, peers of other mothers with similar conditions with whom to compare and mirror. Redefining self-identity was both complex and integral to healing for the women in our study who had multiple triggers for biographical disruption and loss of self. Having cardiac disease amplifies the physical, emotional and sociological vulnerability that women experience perinatally and in the postpartum period [12].

## The role of stories

The women in our study largely traversed these experiences and phases without connection with peers and this sense of isolation increased their suffering. When they were able to connect and ‘see themselves in the stories of others’, often through Facebook support groups, they mostly felt validated and re-assured that others had similar symptoms and experiences, and that they were simply still alive. Stories are not inherently positive or even benign, and can exclude as much as they include, as a couple of women in our study experienced when they did not identify with others in the online groups. The key determinant of the nature of a story is who is allowed to narrate it, who is the protagonist, so even in not identifying with other women, that process helped define their own identities. That is, the telling of their stories is more than a retelling of events, rather it can be reparative and facilitate the conception of their new self [60]. The women in our study found several ways to control or at least own their own narrative. They did this by not staying ‘immersed in their illness’, seeking improved communication, providing others support, advocacy, and involvement in research [61]. As they redefined and clarified their self-identity they shifted from being a ‘cardiac patient’ or an ‘obstetric patient’ to being a woman and mother who juggles having a cardiac condition along with all the other roles and tasks that she needs to do each day.

## Recovery

Regaining a sense of control and autonomy was an important step in recovery and this was underpinned by their sense of self. Recovery is not taken as returning to one’s premorbid condition or to cure, rather it is about recalibrating and finding equilibrium; having a chronic illness means having to adjust and re-adjust, developing new ways to manage for life [56].

Recovery for the women was multi-factorial and non-linear. It was comprised of a partial return to their previous health and life and a partial creation of a new identity and life, now with a chronic health condition and a baby. Our study is consistent with previous research that found that women struggle to recover psychologically, that little or no professional mental health support was provided and that women felt invisible and isolated during recovery [21]. Of note, Koutrolou-Sotiropoulou, Lima, and Stergiopoulos found that more than half of women with PPCM did not return to premorbid levels of emotional health after 1 year despite 68% having recovered cardiac function, highlighting the persistent emotional toll of CDPP on young mothers [62].

## Limitations and strengths

A strength of this study is that it is the first study exploring women’s lived experiences across a spectrum of CDPP, enabling the inclusion of women with rare diseases who otherwise may not be included in research due to small numbers. The interviews facilitated women in being able to be authentic and share what was of most importance to them. The themes developed provide rich and detailed analysis of women’s experiences. This knowledge contributes valuable information to a small body of knowledge on women’s experiences and values relating to CDPP.

The majority of women with CDPP interviewed in our study had not debriefed their experiences, or told their story, even to themselves. The opportunity to narrate unscripted was enlightening for many of them as they reflected upon their own distress and recovery, and clarified their goals for themselves and future girls and women. In this way, having a voice and telling their story was part of their self-mastery and enabled them to (re-)construct their life story retrospectively, in the moment, and prospectively [63]. Writing your own story also allows for the possibility of turning negative experiences into positive ones.

Methodological limitations include that the narratives may be subject to recall bias, both positive and negative, as time has passed since the experiences discussed. Further, the generalisability of our findings is limited to English-speaking patients with no representation of Australian First Nations women or minority ethnicities; no interviewees were in same-sex relationships and there was no inclusion of women living with disability. The majority of interviewees responded to social media recruitment strategies and thus women not using social media are unlikely to be included in this study. Whilst including a range of cardiac diseases is a strength, it is also a limitation, in particular when consideration is to be given to responding the needs identified. More studies are needed to understand specific needs of women with CDPP, including the needs of diverse populations and changing needs over time.

## Conclusion

Acknowledging and understanding the breadth, complexity and depth of women’s experiences of CDPP is a fundamental step in improving outcomes. These findings provide a unique insight into women’s experiences and challenges across a spectrum of diseases and timing of disease. Most women in this study did not report isolated or singular trauma or distressing event, rather there was a layering of traumatic experiences, and the number, nature and recovery from previous traumas informed and complicated the trauma associated with their cardiac disease in pregnancy and the postpartum period. The physical, quality of life and mental health impacts of CDPP are long-term and enhanced continuity of care beyond the routine 6-week postpartum check is essential.

It is recommended that mental health screening timeframes reflect the research findings of both antenatal and late postpartum mental health prevalence and include prior history. Early assessment and treatment increase the chance of improved short- and long-term mental health and cardiac outcomes. In addition, it is essential that mental health screening for women with CDPP is broader than routine perinatal depression screening, includes anxiety, depression, PTSD and other mental health disorders, and is integrated in care pathways. Further research is required to understand long-term outcomes and to refine the findings for specific disease cohorts to be able to provide services and support that meet the needs and values of women with cardiac disease in pregnancy and the first year postpartum.

## Data Availability

Research data are not shared. The data are not available due to privacy or ethical restrictions. Please direct any enquiries to Jane Hutchens.

## Abbreviations

CDPP: Cardiac disease in pregnancy and postpartum
ED: Emergency department
NICU: Neonatal intensive care unit
NSW: New South Wales
PPCM: Peripartum cardiomyopathy
PSCAD: Pregnancy associated spontaneous coronary artery dissection
PTSD: Post-traumatic stress disorder
SCAD: Spontaneous coronary artery dissection
W: Woman
